# An aposymbiotic primary coral polyp counteracts acidification by active pH regulation

**DOI:** 10.1038/srep40324

**Published:** 2017-01-18

**Authors:** Yoshikazu Ohno, Akira Iguchi, Chuya Shinzato, Mayuri Inoue, Atsushi Suzuki, Kazuhiko Sakai, Takashi Nakamura

**Affiliations:** 1 Marine and Environmental Sciences Course, Graduate School of Engineering and Science, University of the Ryukyus, Senbaru 1, Nishihara, Okinawa 903-0213, Japan; 2 Department of Bioresources Engineering, National Institute of Technology, Okinawa College, 905 Henoko, Nago, Okinawa 905-2192, Japan; 3 Marine Genomics Unit, Okinawa Institute of Science and Technology Graduate University, Onna, Okinawa 904-0495, Japan; 4 Graduate School of Natural Science and Technology, Okayama University, 3-1-1 Tsushima-naka, Okayama 700-8530, Japan; 5 Geological Survey of Japan, National Institute of Advanced Industrial Science and Technology (AIST), 1-1-1 Higashi, Tsukuba, Ibaraki 305-8567, Japan; 6 Sesoko Station, Tropical Biosphere Research Center, University of the Ryukyus, 3422 Sesoko, Motobu, Okinawa 905-0227, Japan; 7 Japan Science and Technology Agency (JST)/Japan International Cooperation Agency (JICA) SATREPS, Tokyo, Japan

## Abstract

Corals build their skeletons using extracellular calcifying fluid located in the tissue–skeleton interface. However, the mechanism by which corals control the transport of calcium and other ions from seawater and the mechanism of constant alkalization of calcifying fluid are largely unknown. To address these questions, we performed direct pH imaging at calcification sites (subcalicoblastic medium, SCM) to visualize active pH upregulation in live aposymbiotic primary coral polyps treated with HCl-acidified seawater. Active alkalization was observed in all individuals using vital staining method while the movement of HPTS and Alexa Fluor to SCM suggests that certain ions such as H^+^ could diffuse via a paracellular pathway to SCM. Among them, we discovered acid-induced oscillations in the pH of SCM (pH_SCM_), observed in 24% of polyps examined. In addition, we discovered acid-induced pH up-regulation waves in 21% of polyps examined, which propagated among SCMs after exposure to acidified seawater. Our results showed that corals can regulate pH_SCM_ more dynamically than was previously believed. These observations will have important implications for determining how corals regulate pH_SCM_ during calcification. We propose that corals can sense ambient seawater pH via their innate pH-sensitive systems and regulate pH_SCM_ using several unknown pH-regulating ion transporters that coordinate with multicellular signaling occurring in coral tissue.

Coral calcification is believed to occur in a physiologically controlled environment in the extracellular calcifying fluid located in the tissue–skeleton interface [subcalicoblastic medium (SCM)]^1^. The conditions in calcifying fluid during active calcification are assumed to be very different from those of the external seawater environment^2^,^3^,^4^. The calicoblastic epithelial cells surrounding the SCM are believed to elevate the pH in the SCM (pH_SCM_), facilitate the conversion of bicarbonates to carbonates, and increase the aragonite saturation state (Ω_arag_) followed by facilitation of skeletal growth at the site of calcification. However, the physicochemical conditions in the SCM (e.g., ionic composition) are largely unknown.

Calcification generates protons in the SCM via the chemical reaction Ca^2+^ + HCO^3−^ → CaCO_3_ + H^+3^. The active removal of protons from the SCM leads to higher pH_SCM_ (approximately pH_NBS_: 8.6–10.0)^1^,^4^ and facilitates calcification^3^. Ocean acidification, which is driven by seawater uptake of carbon dioxide (CO_2_) and is recognized as a severe threat to calcifying organisms^5^, causes not only a decrease in the pH of the exterior seawater followed by that in the Ω_arag_, but also decreases in the pH_SCM_ and calcification^6^, suggesting that exterior seawater and calcifying fluid are strongly linked by a paracellular pathway (i.e. ions from seawater are able to enter the calcifying fluid directly, without transiting through the cytoplasm)^7^,^8^,^9^,^10^. Primary polyps of *Favia fragum* reared under very low Ω_arag_ conditions (Ω_arag_ = 0.22) were able to create aragonite crystals beneath the polyp^11^. Additionally, pH measurements of the calcifying fluid of corals using boron isotopes showed that the pH of the extracellular calcifying fluid is higher than that of seawater and systematically decreases with seawater pH (0.3–0.6 units)^12^. These studies suggested that corals could remove protons (upregulate pH) in the SCM in acidified seawater, given that the decrease in pH at calcification sites was not steep relative to that of seawater.

The use of boron isotopes has provided details about the pH conditions for calcification, but this usage relies on an indirect geochemical technique using a boron isotope pH proxy and cannot reveal the mechanisms of pH upregulation by corals at calcification sites^12^,^13^. By contrast, it is worth noting that an *in vivo* pH imaging method using live coral tissues permits direct pH measurements at calcification sites, providing detailed information of pH dynamics in coral tissue^1^,^6^. Applying this advanced physiological technique will facilitate deeper understanding both of the physiological characteristics of the SCM and the manner in which the pH_SCM_ during coral calcification responds to ambient seawater conditions, such as changes seawater pH^6^.

To study the regulation of pH_SCM_, we performed a new time-lapse pH imaging method monitoring change in pH of the calcifying fluid during seawater acidification^7^,^8^,^9^,^10^. Acidified seawater was prepared by using HCl in this experiment because we aimed to monitor acid-induced pH upregulation in coral. The physiological responses of marine calcifiers (e.g., coccolithophores^14^, sea urchin and bivalve^15^) in HCl-acidified seawater are not strictly similar to that in the situation of CO_2_-driven ocean acidification from the aspect of marine CO_2_ chemistry. However, we recognize that our experimental results would help us to understand the physiological responses of corals to acid stress such as daily changes in seawater pH and near-future ocean acidification. We discovered novel pH upregulation, acid-induced oscillations of pH_SCM_, and occasional acid-induced pH upregulation waves (velocity: 1–6 μm/s), which propagated among the SCMs induced by surrounding seawater acidification. In the present study, we have shown that corals can regulate pH_SCM_ more dynamically than was previously believed, which would be regulated by several types of pH-regulating ion transporters that coordinate with multicellular signaling in coral tissue.

## Results and Discussion

### Visualization of skeletal formation of aposymbiotic primary coral polyps on a glass-based dish

**Figure 1:**
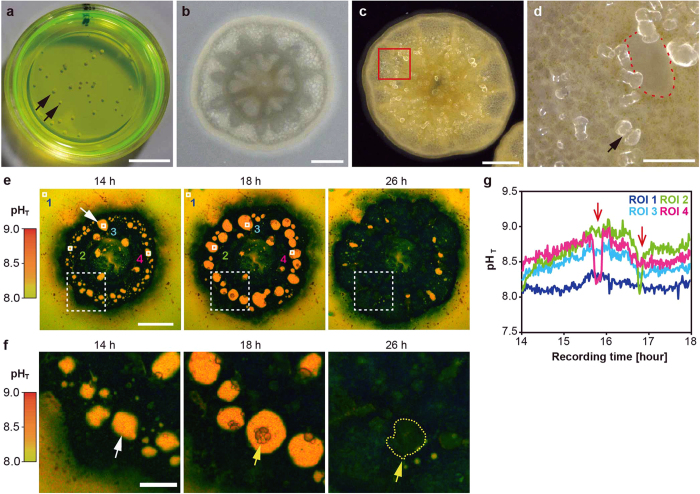
Continuous pH imaging of a primary polyp in HPTS. (**a**) Coral primary polyps in a glass-based dish under HPTS incubation (green colored solution). Black arrows show individual polyps. (**b**) A whole image of a coral primary polyp from the top. (**c**) An image of a coral primary polyp under a dark microscopy field from the bottom. The area enclosed by the red square corresponds to that shown in (**d**). (**d**) A high-magnification image of the enclosed area from (**c**). The black arrow indicates a crystal. The aperture area enclosed by a red dotted line shows presumable subcalicoblastic medium (SCM). (**e**) The numbers in the upper parts of the panels indicate recording times. Time series of pseudocolor images indicate the distribution of pH values (total pH scale in left parts) in developmental process of the primary polyp at the bottom (14–26 h). The white arrow indicates the SCM (orange) and black regions correspond to coral tissues. ROIs 1–4 were examined for intensity changes. Scale bar: 200 μm. Also see Supporting Movie 1. (**f**) A high-magnification image of the white-dashed line square in (**e**). The white arrow indicates the SCM, and yellow arrows and dashed lines the developing crystals (14–26 h). Scale bar, 100 μm. (**g**) pH_SCM_ changes in ROIs 1–4 over time (14–18 h). Red arrows show the sudden decrease of the pH_SCM_.

We used aposymbiotic primary polyps of *Acropora digitifera*^16^, which can be easily prepared with the metamorphosis-inducing peptide Hym-248^17^ in a glass-based dish (Fig. 1a, black arrows). Primary polyps without symbiotic algae (Fig. 1b) began calcification shortly after larval settlement with skeletal formation of CaCO_3_ structures (Fig. 1c,c: black arrow). Because the skeletal growth of aposymbiotic primary polyps of *A. digitifera* is very sensitive to exposure to acidified seawater^18^, this system is convenient for observing changes in pH_SCM_ after exposure to acidified seawater. Calcification occurs at the interface between the tissue and the glass substrate^1^,^6^. Our system allowed the visualization of the coral primary polyp SCM (Fig. 1d: red dotted line) which could estimate pH_SCM_ without the effects of photosynthesis and respiration by symbiotic algae^18^,^19^.

### Continuous pH imaging during coral calcification

To investigate whether coral primary polyps upregulate pH at calcification sites, we applied time-lapse pH imaging (1 min time-interval and 12-h observation) of SCM during calcification. We used 8-hydroxypyrene-1,3,6-trisulfonic acid (HPTS)^20^ (Fig. 1a), a highly water-soluble compound with low toxicity that cannot penetrate the cell membrane^21^, as a pH-sensitive fluorescent indicator (Fig. S1). High concentrations of HPTS (1 mM) were diluted continuously in the seawater to enhance the fluorescent signals from the extracellular space (the SCM) and to offset the autofluorescence of primary polyp tissues.

Coral primary polyps without symbiotic algae of *A. digitifera* had the ability to alkalize their SCMs (Fig. 1e,f: white arrows, pseudocolor in orange), relative to the pH of ambient seawater (pH 8.1, pseudocolor in yellow). Alkalization of the SCMs is expected to correlate with increases in carbonate ion (CO_3_^2−^) concentration and to promote the precipitation of CaCO_3_^1^,^3^,^6^. Initial crystals emerged (Fig. 1f: yellow arrows) and gradually developed at the location of the SCMs (Fig. 1f: yellow dotted lines) during continuous pH imaging. However, 26 h after incubation, most SCMs disappeared and SCM areas were replaced with crystals (Fig. 1f; see also Supporting Movie 1). Thus, long-term incubation combining HPTS pH measurements is applicable to monitoring pH_SCM_ dynamics without major effects on normal developmental processes in aposymbiotic coral primary polyps.

Spatial variations of pH_SCM_ and calcification sensitivity to acidified seawater were observed in adult *Stylophora pistillata*^22^. In our study, the different pH_SCM_ values in some defined regions of interest (ROIs, Fig. 1e: white frames) and periodic fluctuations in pH_SCM_ were detected even within primary polyps of *A. digitifera* examined (Fig. 1g). The observed range of alkalization relative to the seawater (average ± S.D. measured for 241 points) for ROI 2, 3, and 4 were 8.67 ± 0.21, 8.47 ± 0.12, and 8.60 ± 0.15, respectively. At the start of the experiment, the pH_SCM_ values were different between ROIs (Fig. 1g: ROI 2–4) and they were gradually alkalized 15 h after metamorphosis. Surprisingly, sudden drops in pH were observed (0.5–0.8 pH units; Fig. 1g: ROI 2 and 4, red arrows), after which the pH increased within 30 min. We cannot explain this phenomenon, but we speculate that primary polyps temporarily stopped the alkalization of pH_SCM_ or discharged acidic compounds^3^,^23^,^24^,^25^ (such as coral acid-rich proteins) into the SCMs.

### Active pH upregulation of SCM induced by acidified seawater

The mechanisms behind active pH_SCM_ upregulation in corals are unknown. To visualize the dynamics of pH_SCM_ with changes in ambient seawater pH, we induced pH_SCM_ changes by adding HCl- acidified seawater (*n* = 19). Acidified HPTS containing seawater was labeled using Alexa Fluor^®^ 568 dye to simultaneously monitor the putative diffusing H^+^ into SCM via the paracellular pathway. Alexa Fluor^®^ 568 dye is the red fluorescent anionic compounds (10,000 MW in size) which cannot pass through the cell membrane and have no overlapping fluorescence with HPTS for pH measurements in our experimental system (Fig. S1g,h).

**Figure 2:**
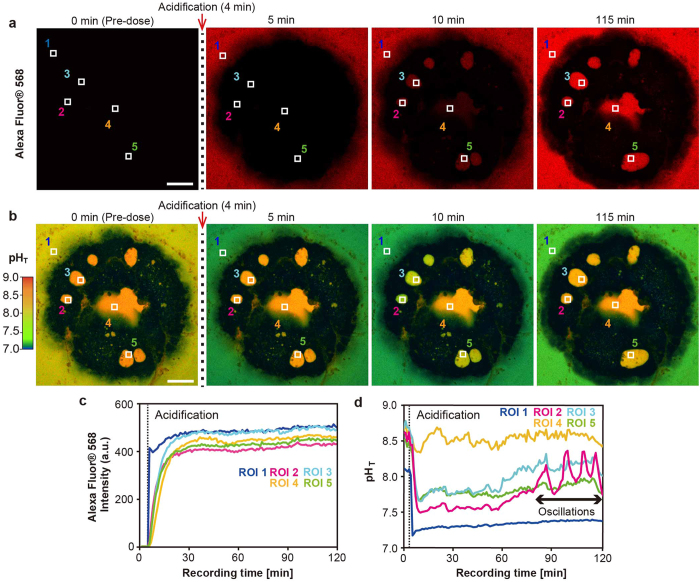
Dynamic pH_SCM_ upregulation induced by acidified seawater observed by pH imaging. (**a**) Acidified seawater was added 4 min after the start of the experiment. Numbers in upper parts of panels indicate recording times. The distribution of Alexa Fluor^®^ 568-labeled acidified seawater was visualized as a red color soon after addition of acidified seawater (containing Alexa Fluor^®^ 568 and HPTS). ROIs 1–5 were examined for intensity changes in (**c**). Scale bar: 100 μm. Also, see Supporting Movie 2. (**b**) Time series of images showing continuous pH imaging after adding acidified seawater. ROIs 1–5 were set for pH changes in d. Scale bar, 100 μm. Also, see Supporting Movie 3. (**c**) Fluorescence intensity changes in five ROIs in arbitrary units over time. The time point of addition of acidified seawater is indicated by a black dashed line in panels in (**c**,**d**). (**d**) pH_SCM_ changes in acidified seawater conditions. pH oscillations were detected 90 min after addition of acidified seawater.

Four minutes after the start of the experiment, acidified seawater was added and red fluorescence from Alexa Fluor^®^ 568 was detected in ambient seawater 1 min later, but no red fluorescence was observed in the SCMs (Fig. 2a: 5 min; see also Supporting Movie 2). When ambient seawater was acidified (to approximately pH 7.2), the seawater turned green in color in pH imaging (Fig. 2b: 5 min; see also Supporting Movie 3), but the pH_SCM_ remained unchanged. Ten minutes after the start of the experiment, Alexa Fluor^®^ 568 continuously flowed into SCMs (Fig. 2a,b: 10 min), and the red signal was detected from SCMs within approximately 20 min (Fig. 2c), suggesting that H^+^ could diffuse via a paracellular pathway^7^,^8^,^9^,^10^. However, the decrease in pH_SCM_ stopped within 5 min of addition of acidified seawater, and the pH_SCM_ was then maintained at a higher value than that of the ambient seawater (Fig. 2d). Approximately 1 pH unit upregulation was detected in all of the primary polyps after exposed to acidified seawater (*n* = 19).

### Acid-induced pH oscillations and pH wave in an aposymbiotic primary polyp

Interestingly, acid-induced oscillations in pH_SCM_ (hereafter called pH oscillations) were detected approximately 90 min after the start of the experiment in this individual polyp (Fig. 2d: ROI 2). The oscillation amplitudes were 0.3–0.6 pH units at approximately 10-min intervals, but clear pH oscillations, like those observed for ROI 2, were not detected in the other ROIs. Although slight pH fluctuations (<0.3 pH units) were detected for ROI 3–5 after adding acidified seawater, they were too small to be evaluated. The pH oscillations were detected in five of the 19 polyps, but no significant change in pH_SCM_ was detected except under acidified seawater conditions (*n* = 3; Fig. S1g). Thus, we concluded that the acid-induced pH oscillations described above were a biological response of corals exposed to acidified seawater. This finding suggests the existence of an unknown pH regulation mechanism induced by the continuous diffusing H^+^ into the SCMs (Fig. 2c). Such an acid-induced biological response (<2 h) has not yet been reported in corals, although cellular mechanisms in response to alterations in external pH have been examined in many other organisms. For example, a sour taste is detected immediately by taste receptor cells that respond to acids in human beings^26^. Even bacteria such as *Escherichia coli* can recover their cytoplasmic pH within 2 min after exposure by acid stress^27^. In order to understand the detailed mechanism of acid-induced biological responses in corals, ion transporters mediating pH regulation in calicoblastic cells should be investigated in the near future^28^,^29^.

**Figure 3:**
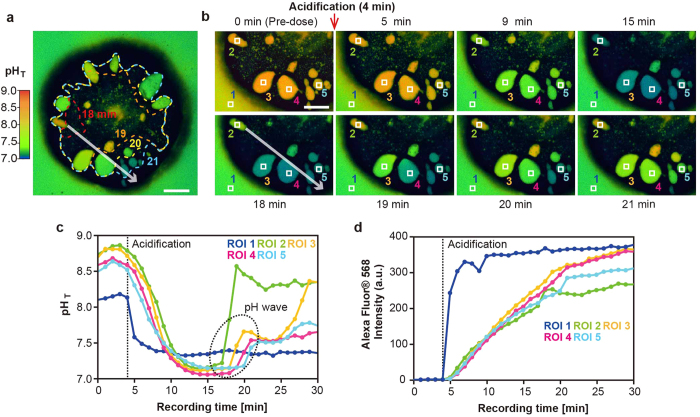
Acid-induced pH wave in a coral primary polyp observed by pH imaging. (**a**) The image shows the primary polyp 20 min after the start of recording. The white arrow shows the direction of the propagating pH wave among the SCMs in the primary polyp. The shape of a wave at a given time point (min) is depicted by the dashed lines. Scale bar, 100 μm. Also, see Supporting Movie 4. (**b**) Time course of pH_SCM_ changes during the experiment at the lower left of the individual polyp in (**a**). The numbers in the upper and bottom parts of the panels indicate the recording times. ROIs 1–5 were examined for intensity changes in (**c**). The white arrow indicates the direction of the propagating pH wave that corresponds to the one observed in (**a**). The red arrows indicate the wavefront of pH upregulation. (**c**) pH_SCM_ changes in the five ROIs defined in (**b**). The circle in black dash lines indicates serialized pH_SCM_ upregulation in ROIs 2–5 (i.e., wave fronts of pH upregulation). (**d**) Alexa Fluor^®^ 568 signal changes in five ROIs in arbitrary units (AUs) over time set at the same position as in (**b**).

We also discovered an acid-induced pH regulation wave (hereafter, a pH wave) in four of the 19 coral primary polyps. To characterize the behavior of the pH regulation waves, we evaluated a representative case of polyp tissue in which a pH wave crossed from the upper left to the lower right (Fig. 3a: white arrow) at 3-min intervals (Fig. 3a; see also Supporting Movie 4). We found that the velocity of the pH wave was approximately 1–6 μm/s in this primary polyp. Four ROIs (2–5) in SCMs and ROI 1 in ambient seawater were defined in the direction of wave propagation (Fig. 3b: white arrow), and a pH wave appeared to increase the pH_SCM_ as it moved in this direction, as shown by the white arrow (Fig. 3b; also see area encircled in black dashed lines in Fig. 3c).

We assume that the pH wave propagation and pH oscillation observed in corals might be mediated by intercellular calcium signaling, given that cytosolic calcium signaling is a general phenomenon of signaling in excitable and non-excitable cells^30^,^31^,^32^, including corals^3^,^28^,^29^,^33^. The velocity of the pH wave was similar to fast calcium waves^33^,^34^,^35^ mediated by Ca^2+^ channels, Ca^2+^ pumps, and GAP junctions, that are major components of the human ion trafficking system and have also been identified in the coral genomic data^34^,^36^. Furthermore, a study using the hippocampal neurons showed that electrical stimulation induced intracellular pH and Ca^2+^ oscillations simultaneously^37^. Thus, the general framework of pH regulation in SCMs (pH_SCM_ alkalization and pH_SCM_ response to ambient seawater) is presumably coordinated by multicellular calcium signaling throughout the calicoblastic cells. These multicellular cell-to-cell communication may play a significant role in balancing the pH homeostasis of entire calcifying tissue during the skeletal formation and growth. The pH_SCM_ alkalization can be partly explained by the activation of ATP-driven Ca^2+^/H^+^ antiporters (i.e., Ca^2+^-ATPase) located at the plasma membrane^3^,^38^. However, it is unknown whether the regulation of pH_SCM_ (pH sensing, pH oscillation, and the pH wave observed in this study) can be achieved only via activation of Ca^2+^-ATPase.

Despite the ambient seawater pH decrease to approximately 7.4, more extreme pH_SCM_ decrease (0.1–0.3 pH units from the ambient seawater pH) were observed 15 min after the start of the experiment (in five of the 19 polyps; Fig. 3b,c). In addition, the pH_SCM_ in ROI 2 considerably increased (1.4 pH unit) within a 2-min interval (17–19 min after the start of the experiment) and suddenly stopped alkalization at 20 min (Fig. 3c: ROI 2), while continuous H^+^ diffusion into SCMs. These dynamic pH_SCM_ upregulations are noteworthy. To further understand the mechanism underlying pH_SCM_ upregulation in corals, we must focus on pH-sensitive ion channels^39^, given that the presence in the *A. digitifera* genome of well-known pH-sensitive ion channels (e.g., transient receptor potential channels, acid-sensing channels, and chloride channels) has been reported^34^,^36^. Other ion transporters such as bicarbonate ion transporters^40^ or carbonic anhydrase^41^ might be indispensable for the alkalization and supply process of dissolved inorganic carbon at calcification sites^4^,^42^. Further experiments should be needed for understanding the pH regulation mechanism in corals.

In summary, we found that corals can regulate pH_SCM_ more dynamicaly than they were previously believed, suggesting that pH-sensing and -regulating mechanisms underlie coral calcification. We propose that several types of pH-sensitive ion transporters regulate alkalization of SCM in a coordinated manner. Understanding the pH upregulation of corals observed in this study will be essential for predicting how they regulate pH at the calcification site and how they respond to ongoing ocean acidification. In addition, our observations suggest not only that pH_SCM_ dynamics were regulated by surrounding calicoblastic epithelium cells but also that the pH among SCMs was coordinated at the tissue level via long-range multicellular signaling beneath the primary polyp. These findings pave the way for future studies of the multicellular regulation mechanisms involved in intercellular calcium signaling during skeletal formation. However, we could not clearly observe intracellular calcium signals because of the difficulty of monitoring these signals in the presence of autofluorescence from coral tissue or staining of calicoblastic epithelium cells with fluorescent calcium indicators^31^,^32^. In future, it is crucial to investigate the physiological mechanisms proposed above to analyze the physiological adaptation and cellular response of corals to environmental disturbances such as ocean warming, ocean acidification, and eutrophication.

## Methods

### Sample preparation

The scleractinian coral *A. digitifera*, which is one of the most common species in the Ryukyu Islands of Japan^43^, was used. Gravid colonies of *A. digitifera* were collected from a fringing reef at Sesoko Island, Motobu-cho, Okinawa, Japan. The colonies were kept in a running seawater tank under natural light conditions at Sesoko Station, Tropical Biosphere Research Center, University of the Ryukyus, Okinawa, Japan. Coral spawning occurred at night around the time of the full moon in the spring and summer seasons of 2015. Gametes were collected after spawning, as described by Inoue *et al*.^19^ Primary polyps were prepared by induction of the settlement of planula larvae (3–30 days old) using the coral metamorphosis inducer peptide Hym-248^17^. Hym-248 induces the synchronous metamorphosis and settlement of *Acropora* planulae and is a useful tool for studies of *Acropora* larval metamorphosis. Approximately 10–20 larvae were placed in a glass-based dish (No. 1S, thickness: 0.15–0.18 mm; IWAKI Glass, Tokyo, Japan) with 40 μL of filtered seawater (FSW: pore size 0.22 μm). Next, a 10-μL aliquot of 2 × 10^−4^ M Hym-248 in FSW was added to the dish and the larvae were incubated for 2 h to induce metamorphosis. Larvae that settled at the seawater surface and on the side of the dish were removed. The pH of the seawater was measured with a portable pH meter (D-71, Horiba Ltd., Kyoto, Japan) against a total hydrogen ion concentration scale^44^,^45^. A stock solution of 10-mM HPTS (Sigma-Aldrich, St. Louis, MO, USA) was prepared and the pH was adjusted to 8.1 (Total scale: pH_T_) using sodium hydroxide (NaOH). This solution was then diluted with FSW and buffered to pH 8.1 with NaOH to obtain a final concentration of 1 mM (FSW-HPTS: salinity of approximately 35). After a 2-h incubation period with Hym-248, the solution was made to a volume of 2000 μL with FSW–HPTS. The specimens were incubated with FSW–HPTS in an incubator (VERSOS, Hiroshima, Japan) in the dark at 27.0 ± 0.1 °C prior to pH imaging. Bright-field, dark-field, and polarization images were acquired with a Keyence VHX-2000 digital microscope (Osaka, Japan).

### pH imaging

We used an inverted confocal imaging system (A1+ confocal microscope system; Nikon Instruments Inc, Tokyo, Japan) equipped with a high-resolution galvano scanner and operated with NIS Elements software (Nikon Instruments Inc. Tokyo, Japan). A CFI Plan Apo × 10 objective lens (NA 0.45, Nikon) was used to capture the whole image of the bottom of the coral tissue. The screen display resolution for viewing confocal microscope images was 1024 × 1024 pixels (0.86 μm = 1 pixel). Primary polyps at 14–18 h after metamorphosis were placed on a glass-based dish at room temperature (27 ± 0.2 °C). An acid-induced biological response in pH_SCM_ was induced with acidified HPTS solution containing Alexa Fluor^®^ 568 (Thermo Fisher Scientific, Waltham, MA, USA). Before the start of the experiment, 1000 μL of FSW–HPTS was gently extracted. During pH imaging, a mixed solution of 1000 μL of acidified HPTS (1 mM, pH 7.0–7.2) and Alexa Fluor^®^ 568 (20 μM) was added 4 min after the start of recording to label the adding acidified FSW–HPTS. The dual-excitation ratiometric indicator HPTS was excited at wavelengths of 405 and 440 nm, and fluorescence was detected at 510–530 nm. Alexa Fluor^®^ 568 was excited at 561 nm and fluorescence was detected at 610–640 nm 1 s after HPTS fluorescence was detected. Intervals were 1 min apart. pH calibration was performed by determining the ratio of HPTS fluorescence in FSW containing 1-mM HPTS adjusted with HCl and NaOH to the range of pH 6–9 at 25.0 ± 0.1 °C (Fig. S1). We could not find spectral shift of HPTS between 25 °C and 27 °C using spectro-photometer (Jasco Corporation, Tokyo, Japan). Ratio images that fitted the pH values were created using NIS Elements software. ImageJ (Rasband, WS. ImageJ. US National Institutes of Health, Bethesda, Maryland, USA. http://imagej.nih.gov/ij/; 1997–2014) was used to measure the velocity of pH waves.

## Additional Information

**How to cite this article**: Ohno, Y. *et al*. An aposymbiotic primary coral polyp counteracts acidification by active pH regulation. *Sci. Rep.* **7**, 40324; doi: 10.1038/srep40324 (2017).

**Publisher's note:** Springer Nature remains neutral with regard to jurisdictional claims in published maps and institutional affiliations.

## Supplementary Material

Supplementary Information

Supplementary Video 1

Supplementary Video 2

Supplementary Video 3

Supplementary Video 4

## References

[b1] Venn A., Tambutté E., Holcomb M., Allemand D. & Tambutté S. Live tissue imaging shows reef corals elevate pH under their calcifying tissue relative to seawater. Plos One 6, e20013 (2011). 21637757 10.1371/journal.pone.0020013 PMC3103511

[b2] Tambutté E. . Observations of the tissue-skeleton interface in the scleractinian coral *Stylophora pistillata*. Coral Reefs. 26, 517–529 (2007).

[b3] Erez J., Reynaud S., Silverman J., Schneider K. & Allemand D. Coral calcification under ocean acidification and global change, p. 151–176. In Dubinsky Z. & Stambler N. [eds.], Coral reefs: An ecosystem in transition. Springer, Dordrecht (2011).

[b4] Wei-Jun Cai. . Microelectrode characterization of coral daytime interior pH and carbonate chemistry. Nat. Commun. 7, 11144 (2016). 27041668 10.1038/ncomms11144 PMC4821998

[b5] Hoegh-Guldberg O. . Coral reefs under rapid climate change and ocean acidification. Coral reefs under rapid climate change and ocean acidification. Science. 318, 1737–1742 (2007). 18079392 10.1126/science.1152509

[b6] Venn A. A. . Impact of seawater acidification on pH at the tissue-skeleton interface and calcification in reef corals. Proc. Natl. Acad. Sci. USA 110, 1634–1639 (2013). 23277567 10.1073/pnas.1216153110 PMC3562847

[b7] Cohen A. L. & McConnaughey T. A. Geochemical perspectives on coral mineralization. Rev. Mineral. Geochem. 54, 151–187 (2003).

[b8] Tambutté E. . Calcein labelling and electrophysiology: insights on coral tissue permeability and calcification. Proc. Biol. Sci. 279, 19–27 (2012). 21613296 10.1098/rspb.2011.0733 PMC3223652

[b9] Gagnon A. C., Adkins J. F. & Erez J. Seawater transport during coral biomineralization. Earth Planet. Sci. Lett. 329, 150–161 (2012).

[b10] Inoue M. . Controlling factors of Ca isotope fractionation in scleractinian corals evaluated by temperature, pH and light controlled culture experiments. Geochim. Cosmochim. Acta. 167, 80–92 (2015).

[b11] Cohen A. L., McCorkle D. C., de Putron S., Gaetani G. A. & Rose K. A. Morphological and compositional changes in the skeletons of new coral recruits reared in acidified seawater: Insights into the biomineralization response to ocean acidification. Geochemistry, Geophys. Geosystems. 10, Q07005 (2009).

[b12] McCulloch M., Falter J., Trotter J. & Montagna P. Coral resilience to ocean acidification and global warming through pH up-regulation. Nat. Clim. Chang. 2, 623–627 (2012).

[b13] Gagnon A. C. Coral calcification feels the acid. Proc. Natl. Acad. Sci. USA 110, 1567–1568 (2013). 23329329 10.1073/pnas.1221308110 PMC3562801

[b14] Rost B., Zondervan I. & Wolf-Gladrow D. Sensitivity of phytoplankton to future changes in ocean carbonate chemistry: Current knowledge, contradictions and research directions. Mar. Ecol. Prog. Ser. 373, 227–237 (2008).

[b15] Kurihara H. Effects of CO_2_-driven ocean acidification on the early developmental stages of invertebrates. Mar. Ecol. Prog. Ser. 373, 275–284 (2008).

[b16] Hirose M., Yamamoto H. & Nonaka M. Metamorphosis and acquisition of symbiotic algae in planula larvae and primary polyps of *Acropora* spp. Coral Reefs. 27, 247–254 (2007).

[b17] Iwao K., Fujiwara T. & Hatta M. A cnidarian neuropeptide of the GLWamide family induces metamorphosis of reef-building corals in the genus Acropora. Coral Reefs. 21, 127–129 (2002).

[b18] Ohki S. . Calcification responses of symbiotic and aposymbiotic corals to near-future levels of ocean acidification. Biogeosciences. 10, 6807–6814 (2013).

[b19] Inoue M. . Estimate of calcification responses to thermal and freshening stresses based on culture experiments with symbiotic and aposymbiotic primary polyps of a coral, *Acropora digitifera*. Glob. Planet. Change 92, 1–7 (2012).

[b20] de Nooijer L. J., Toyofuku T. & Kitazato H. Foraminifera promote calcification by elevating their intracellular pH. Proc. Natl. Acad. Sci. USA 106, 15374–15378 (2009). 19706891 10.1073/pnas.0904306106 PMC2741258

[b21] Han J. & Burgess K. Fluorescent indicators for intracellular pH. Chem. Rev. 110, 2709–2728 (2010). 19831417 10.1021/cr900249z

[b22] Holcomb M. . Coral calcifying fluid pH dictates response to ocean acidification. Sci. Rep. 4, 5207 (2014). 24903088 10.1038/srep05207 PMC4047535

[b23] Takeuchi T., Yamada L., Shinzato C., Sawada H. & Satoh N. Stepwise evolution of coral biomineralization revealed with genome-wide proteomics and transcriptomics. PLoS One. 11, e0156424 (2016). 27253604 10.1371/journal.pone.0156424 PMC4890752

[b24] Drake J. L. . Proteomic analysis of skeletal organic matrix from the stony coral *Stylophora pistillata*. Proc. Natl. Acad. Sci. USA 110, 3733–3793 (2013). 23431140 10.1073/pnas.1301419110 PMC3593878

[b25] Mass T., Drake J. L., Peters E. C., Jiang W. & Falkowski P. G. Immunolocalization of skeletal matrix proteins in tissue and mineral of the coral *Stylophora pistillata*. Proc. Natl. Acad. Sci. USA 111, 12728–2733 (2014). 25139990 10.1073/pnas.1408621111 PMC4156710

[b26] Chandrashekar J., Hoon M. A., Ryba N. J. P. & Zuker C. S. The receptors and cells for mammalian taste. Nature. 444, 288–294 (2006). 17108952 10.1038/nature05401

[b27] Martinez K. A. . Cytoplasmic pH response to acid stress in individual cells of *Escherichia coli* and *Bacillus subtilis* observed by fluorescence ratio imaging microscopy. Appl. Environ. Microbiol. 78, 3706–3714 (2012). 22427503 10.1128/AEM.00354-12 PMC3346368

[b28] Kaniewska P. . Major cellular and physiological impacts of ocean acidification on a reef building coral. PLoS One 7, e34659 (2012). 22509341 10.1371/journal.pone.0034659 PMC3324498

[b29] Moya a. . Whole transcriptome analysis of the coral *Acropora millepora* reveals complex responses to CO_2_-driven acidification during the initiation of calcification. Mol. Ecol. 21, 2440–2454 (2012). 22490231 10.1111/j.1365-294X.2012.05554.x

[b30] Berridge M. J., Bootman M. D. & Roderick H. L. Calcium signalling: dynamics, homeostasis and remodelling. Nat. Rev. Mol. Cell Biol. 4, 517–529 (2003). 12838335 10.1038/nrm1155

[b31] Ohno Y. & Otaki J. M. Eyespot colour pattern determination by serial induction in fish: Mechanistic convergence with butterfly eyespots. Sci. Rep. 2, 290 (2012). 22375251 10.1038/srep00290 PMC3289039

[b32] Ohno Y. & Otaki J. M. Spontaneous Long-Range Calcium Waves in Developing Butterfly Wings. BMC Dev. Biol. 15, 17 (2015). 25888365 10.1186/s12861-015-0067-8 PMC4445562

[b33] Desalvo M. K. . Differential gene expression during thermal stress and bleaching in the Caribbean coral *Montastraea faveolata*. Mol. Ecol. 17, 3952–3971 (2008). 18662230 10.1111/j.1365-294X.2008.03879.x

[b34] Shinzato C. . Using the *Acropora digitifera* genome to understand coral responses to environmental change. Nature. 476, 320–323 (2011). 21785439 10.1038/nature10249

[b35] Jaffe L. F. Fast calcium waves. Cell Calcium. 48, 102–113 (2010). 20883893 10.1016/j.ceca.2010.08.007

[b36] Bhattacharya D. . Comparative genomics explains the evolutionary success of reef-forming corals. Elife. 5, 1–26 (2016). 10.7554/eLife.13288 PMC4878875 27218454

[b37] Matlashov M. E. . Fluorescent ratiometric pH indicator SypHer2: Applications in neuroscience and regenerative biology. Biochim. Biophys. Acta - Gen. Subj. 1850, 2318–2328 (2015). 10.1016/j.bbagen.2015.08.002 PMC4587288 26259819

[b38] Zoccola D. . Molecular cloning and localization of a PMCA P-type calcium ATPase from the coral *Stylophora pistillata*. Biochim. Biophys. Acta. 1663, 117–126 (2004). 15157614 10.1016/j.bbamem.2004.02.010

[b39] Holzer P. Acid-sensitive ion channels and receptors. In Sensory Nerves. Springer Berlin Heidelberg. 283–332 (2009). 10.1007/978-3-540-79090-7_9 PMC4359897 19655111

[b40] Zoccola D. . Bicarbonate transporters in corals point towards a key step in the evolution of cnidarian calcification. Sci. Rep. 5, 9983 (2015). 26040894 10.1038/srep09983 PMC4650655

[b41] Moya A. . Carbonic anhydrase in the scleractinian coral *Stylophora pistillata*: characterization, localization, and role in biomineralization. J. Biol. Chem. 283, 25475–25484 (2008). 18617510 10.1074/jbc.M804726200

[b42] Allison N., Cohen I., Finch A. A., Erez J. & Tudhope A. W. Corals concentrate dissolved inorganic carbon to facilitate calcification. Nat. Commun. 5, 5741 (2014). 25531981 10.1038/ncomms6741

[b43] Nakajima Y., Nishikawa A., Iguchi A. & Sakai K. Gene flow and genetic diversity of a broadcast-spawning coral in northern peripheral populations. PLoS One 5, e11149 (2010). 20585399 10.1371/journal.pone.0011149 PMC2886843

[b44] Dickson A. G., Sabine C. L. & Christian J. R. (Eds) Guide to best practices for ocean CO_2_ measurements: PICES Special Publication 3. Sidney, Canada: PICES1 (2007).

[b45] Iguchi A. . Effects of acidified seawater on coral calcification and symbiotic algae on the massive coral *Porites australiensis*. Mar. Environ. Res. 73, 32–36 (2012). 22115919 10.1016/j.marenvres.2011.10.008

