# Supplementary material for: An aposymbiotic primary coral polyp counteracts acidification by active pH regulation
Source: Sci Rep. 2017 Jan 18;7:40324. doi: 10.1038/srep40324 (PMC5241827; doi:10.1038/srep40324)
Supplement: Supplementary Information [file srep40324-s1.pdf]

Supplementary information for:

An aposymbiotic primary coral polyp counteracts acidification by active pH regulation

Yoshikazu Ohno[1*], Akira Iguchi[2], Chuya Shinzato[3], Mayuri Inoue[4], Atsushi Suzuki[5], Kazuhiko Sakai[6], and Takashi Nakamura[1, 6, 7]

[1]Marine and Environmental Sciences Course, Graduate School of Engineering and Science, University of the Ryukyus, Senbaru 1, Nishihara, Okinawa 903-0213, Japan
[2]Department of Bioresources Engineering, National Institute of Technology, Okinawa College, 905 Henoko, Nago, Okinawa 905-2192, Japan
[3]Marine Genomics Unit, Okinawa Institute of Science and Technology Graduate University, Onna, Okinawa 904-0495, Japan
[4]Graduate School of Natural Science and Technology, Okayama University, 3-1-1 Tsushima-naka, Okayama 700-8530, Japan
[5]Geological Survey of Japan, National Institute of Advanced Industrial Science and Technology (AIST), 1-1-1 Higashi, Tsukuba, Ibaraki 305-8567, Japan
[6]Sesoko Station, Tropical Biosphere Research Center, University of the Ryukyus, 3422 Sesoko, Motobu, Okinawa 905-0227, Japan
[7]Japan Science and Technology Agency (JST)/ Japan International Cooperation Agency (JICA) SATREPS

[*]Corresponding. yoshikazu.ohno7@gmail.com

Supplementary figure S1

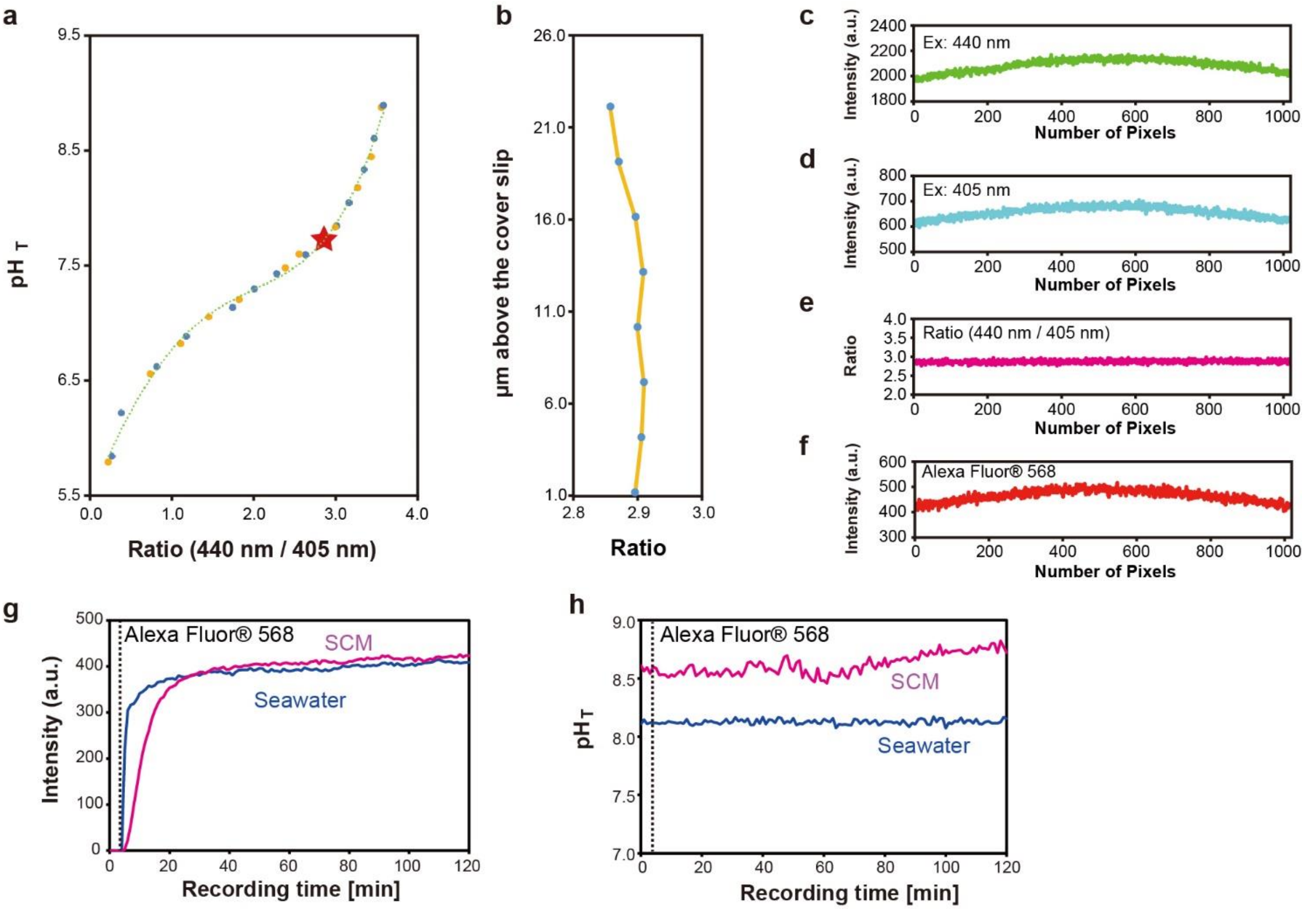

Figure. S1. pH measurement and HPTS calibration. pH calibration was performed at 25.0 ± 0.1°C before (Fig S1a, orange dots) and after (Fig S1a, blue dots) the experimental period in 2015. The pH meter was calibrated with total hydrogen ion concentration pH scale buffers, Tris, and adenosine monophosphate.[41, 42] In the HPTS calibration, background fluorescence values were recorded prior to loading with the dye and subtracted from the values used for pH calculation (background fluorescence values were <5% compared with HPTS fluorescence signals). a**,** The calibration curve (polynomial equation: $y = 0.0272x^4 - 0.0498x^3 - 0.3862x^2 + 1.5984x + 5.6016$, $r = 0.9981$) between the pH and dual-excitation ratio (440 nm/405 nm) of HPTS was determined. (**b–f**) Representative data of HPTS measurement at the point of the red star shown in a. The examples along the short Z-stacks (20 µm) are shown for pH measurements to check whether the pH was stable throughout the area being measured in b. (**c**–**e**) To achieve reliable pH imaging over a wide range of HPTS solutions, we checked the aberration of the objective lens. The sampling line (1024 pixels: 860.64 µm in length; 23 pixels: 19.8 µm in width; 0.86 µm = 1 pixel) was set horizontally in the middle of the HPTS fluorescence images obtained. Examples show that aberrations from the HPTS solution at both 440 nm in c and 405 nm in d excitations were detected, but aberration was not detected in the

value of the fluorescence ratio (440 nm/405 nm) in d. Thus, the quality of our pH imaging system is valid and rigorous for both the pH_{SCM} of the primary polyp and ambient seawater. The pattern of red fluorescence from Alexa Fluor® 568 was also checked in the HPTS solution in f. However, an aberration of the red fluorescence intensity was detected, as shown in c and d. Thus, measuring the liquid concentration using a single excitation is not very accurate in our experimental system. There was no overlapping of the fluorescence between HPTS and Alexa Fluor® 568. (**g, h**) Thus, the control experiment (n = 3) shows no changes in seawater pH and pH_{SCM} after addition of Alexa Fluor® 568 containing seawater (pH_T 8.1). Using this pH imaging method, we were able to simultaneously measure pH_{SCM} (HPTS), ambient seawater pH and diffusing ions to SCM by paracellular pathway (Alexa Fluor® 568).

Movie Legends

Supporting Movie 1. Time-lapse image of pH changes in the SCM
Time series of images show the developmental process of the primary polyp at the bottom (14–26 h). Primary polyps were incubated in HPTS continuously. Black regions correspond to coral tissues because HPTS cannot permeate the cell membrane. 26 h after incubation, most SCMs disappeared and SCM areas were replaced with crystals. Images were collected every 1 minutes. This Video corresponds to data in Figure 1e.

Supporting Movie 2. Diffusion of Alexa Fluor® 568 dye into the SCM
Acidified HPTS containing seawater was labeled using Alexa Fluor® 568 dye. Four minutes after starting the experiment, acidified seawater was added and red fluorescence was detected in ambient seawater after 1 min. Images were collected every 1 min. This Video corresponds to data in Figure 2a.

Supporting Movie 3. Alkalization and pH oscillations in the SCM
pH_{SCM} was maintained at a higher value (yellow) than the ambient acidified seawater (green). pH oscillations in pH_{SCM} (colour of the SCM oscillating between yellow and orange) were detected approximately 90 min after starting the experiment (Figure 2d: ROI 2). Images were collected every 1 minutes. This Video corresponds to data in Figure 2b.

Supporting Movie 4. Rapid alkalization and pH wave in the SCM
Despite the ambient seawater pH decreasing (green), more extreme pH_{SCM} decreases (Blue green) were observed 15 min after starting the experiment (Figures 3b and 3c). 1 After that, a pH wave crossed from the upper left to the lower right. Images were collected every 1 minutes. This Video corresponds to data in Figure 3a and Figures 3b.
